# The validation of a novel method combining both HER2 immunohistochemistry and HER2 dual-colour silver *in situ* hybridization on one slide for gastric carcinoma testing

**DOI:** 10.1186/1479-5876-12-160

**Published:** 2014-06-06

**Authors:** Dominique Werner, Achim Battmann, Kristina Steinmetz, Tobin Jones, Tiffany Lamb, Michele Martinez, Hans-Michael Altmannsberger, Salah-Eddin Al-Batran

**Affiliations:** 1 Institute of Clinical Cancer Research (IKF) at Krankenhaus Nordwest, UCT-University Cancer Center Frankfurt, Steinbacher Hohl 2-26, 60488, Frankfurt/Main, Germany; 2 Institute of Pathology at Krankenhaus Nordwest, Frankfurt am Main, Germany; 3 Roche Pharma, Penzberg, Germany; 4 Ventana Medical System, lnc./Roche Tissue Diagnostics, Tucson, USA

**Keywords:** Gastric cancer, HER2, Immunohistochemistry, Silver in situ hybridization, Gene-protein assay

## Abstract

**Background:**

HER2 status assessment is a prerequisite for the establishment of an appropriate treatment strategy in gastric cancer. Gastric cancers are very heterogeneous and separate evaluations of gene amplification and protein expression lead to uncertainties in localizing distinct clones and are time consuming. This study evaluates the equivalence of the novel method combining both gene and protein platforms on one slide.

**Methods:**

Immunohistochemistry (IHC) and HER2 dual-colour silver in situ hybridization (SISH) as single methods (IHC/SISH) and gene-protein platform combining both methods on one slide (gene/protein) were performed in randomly collected 100 cases of gastric adenocarcinoma. Results of IHC/SISH were compared with gene/protein staining.

**Results:**

96 of 100 samples were assessable. In the gene/protein staining, pathologists were able to assess gene amplification and consequent protein expression at the single cell level. In comparison trials, gene amplification was observed in 14.6% by both, conventional SISH and gene/protein platform (agreement 100%; Kappa-coefficient κ = 1.0). Protein expression scores by IHC were 70.8% (0), 10.4% (1+), 9.4% (2+), and 9.4% (3+). Protein expression by gene/protein method were: 70.8% (0), 11.5% (1+), 7.3% (2+) and 10.4% (3+) of patients. There were complete concordances in IHC assessment of cases with score 0 (100.0%; κ = 1). High concordances are shown in score 1+ (98.96%; κ = 0.947) and 3+ (96.88%; κ = 0.825) cases and good concordances in 2+ cases (95.83%; κ = 0.728).

**Conclusions:**

This novel combined platform has the advantage of being able to evaluate both gene and the protein status in the same cancer cell and may be of particular interest for research and patient’s care.

**Article category:**

Disease Biomarker.

## Background

The HER2 oncogene (also referred to as HER2/neu or ERBB-2) on chromosome 17q21, encodes a 185-kD transmembrane tyrosine kinase receptor that belongs to the epidermal growth factor receptor (EGFR) family comprised of EGFR/HER1, HER2, HER3, and HER4. HER2 activation plays a central role in cell proliferation and survival, largely mediated through the RAS–MAPK pathway. It also inhibits cell death through the phosphatidylinositol 3'-kinase–AKT–mammalian target of rapamycin (mTOR) pathway. HER2 overexpression has been reported in a variety of solid tumors [1-7]. Gene amplifications are rare in other diseases and anti-HER2 therapy is currently validated only in breast and gastric cancers.

Based on the results of the ToGA trial [8], trastuzumab was approved for metastatic adenocarcinoma of the stomach and gastroesophageal junction in combination therapy in the US and Europe. Requirement is proof of HER2 overexpression. In Europe, this is defined by an IHC 3+ result or an IHC 2+ and positive FISH or SISH result (ratio ≥ 2.0). IHC was considered the primary method because IHC negative or weekly positive (1+) patients did not benefit from trastuzumab in the ToGA trial, although they were ISH positive. In the literature, however, there is considerable variation in the immunohistochemical positivity determined. Here, the percentage of HER2 positive advanced gastric adenocarcinomas varies from study to study, from 5% to 30% [9], most likely due to different analysis protocols and interpretation criteria.

Prospective substudies from two of the adjuvant randomized trials of trastuzumab versus nil in breast cancer demonstrated that approximately 20% of HER2 assays performed at on-site institutions (at the primary treatment site’s pathology department) were incorrect when the same specimen was re-evaluated in a high volume, central laboratory [10,11].

Unlike breast cancer, the histopathology of gastric cancer in terms of HER2 is considered to be more heterogeneous and focal overexpression of gene amplification is frequently observed [12]. Therefore, difficulties in determining the HER2 status are expected to be more pronounced than in breast cancer. Overall, taken these results into account, more reliable, reproducible and time sparing methods for assessing the HER2 status in gastric cancer are needed. Only few published studies combining IHC and in situ hybridization methods (but not SISH) on one slide have been conducted [13-16]. Theses studies utilized standard IHC and chromogenic or fluorescence in situ hybridization (CISH or FISH) as single methods and compared them with a simultaneous analysis (combined on one slide) and found good concordance with results of single staining. To the best of our knowledge, however, there are no studies that directly compare results of both immunohistochemistry (IHC) and silver in situ hybridization (SISH) when each is used separately with the results of using them in combination on a single section in gastric carcinomas.

In the present study, the HER2 protein expression and gene amplification status of 100 gastric adenocarcinomas and carcinomas of the esophagogastric junction were examined by conventional IHC and ISH single methods (IHC/SISH) and in parallel by the novel method combining both methods (gene/protein). The aim of this study was to evaluate the equivalence of the new gene-protein platform in comparison to the single staining methods.

## Methods

### Patients and tissue samples

Formalin-fixed paraffin-embedded (FFPE) tumor samples from 100 patients with gastric or gastroesophageal junction adenocarcinoma were selected from a tissue archive of samples at the Institute of Pathology at Krankenhaus Nordwest. The project was performed with the permission of the responsible ethic committee. For each case, four different staining methods were used according to the manufacturer´s FDA-approved procedures: hematoxylin and eosin (H&E) to assess the sample adequacy and to confirm the histological subtype, immunohistochemistry (IHC) to evaluate the HER2 protein expression, silver in situ hybridization (SISH) to evaluate the gene amplification and the novel gene-protein platform which combines IHC and SISH on one slide.

### Immunohistochemistry

IHC was performed with the FDA-approved Ventana PATHWAY rabbit monoclonal antibody 4B5 clone and with the ultraVIEW DAB Detection Kit (Ventana) on a BenchMark XT automated stainer (Ventana, Tucson, AZ). Briefly, the tissue sections were deparaffinized with EZ Prep at 75°C and 76°C, heat pretreated in Cell Conditioning 1 (CC1) for antigen retrieval at 76°C – 100°C and then incubated with the anti-HER2 primary antibody for 16 min at 37°C after inactivation of the endogenous peroxidase using UV-inhibitor for 4 min at 37°C. The slides were incubated with a secondary antibody followed by the application of HRP Universal Multimer for 8 min. Antibodies were detected using chromogen (for 38 min). Before mounting, slides were counterstained with hematoxylin II for 4 min and bluing reagent for 4 min. To support the validity of staining and identify experimental artefacts, a positive control was included in each run.

### Silver in situ hybridization

Dual colour SISH was performed according to the INFORM HER2 Dual ISH DNAProbe cocktail and ultraVIEW SISH DNP Detection Kit/ultraVIEW RED ISH DIG Detection Kit for HER2 and Chr 17 quantitation and procedure (Ventana/Roche). Both probes were labeled with 2,4-dinitrophenol (DNP) and digoxigenin (DIG). The tissue slides were deparaffinized with EZ Prep at 65°C-76°C and heat pretreated with EZ Prep-diluted Cell Conditioner 2 (CC2) at 90°C followed by protease digestion with ISH Protease 3 for 16 min at 37°C. The genomic DNA tissue sections and the nick-translated HER2 and Chr 17 probes were co-denatured by heat treatment for 20 min at 80°C followed by a hybridization step for 6 h at 44°C. The HER2 signals were detected using rabbit anti-DNP antibody for 20 min at 37°C and incubated with a HRP-conjugated goat anti-rabbit antibody for 16 min at 37°C after three 8 min stringency washes. The signal was detected as silver deposits with silver acetate, hydroquinone and hydrogen peroxide. For Chr 17 detection, the slides were incubated with mouse anti-DIG antibody for 20 min at 37°C and with an AP-conjugated goat anti-mouse antibody for 24 min at 37°C. The Chr 17 signal was developed as red dot staining with fast red and naphthol phosphate. The slides were finally counterstained with hematoxylin II and bluing reagent before mounting.

### Gene/protein

Gene/protein platform combining IHC and SISH on one slide. To support the validity of staining and identify experimental artefacts, a positive control was included in each run.For HER2 protein staining and for HER2/Chr 17 hybridization, the iVIEW DAB Detection Kit and ultraVIEW Detection Kits were used. The samples were stained under several of assay conditions to implement an optimum protocol needed to achieve IHC/SISH staining results comparable to those of individual IHC and SISH testing. The optimization was performed in the laboratory of Ventana (Roche) and in part in own work. Optimal results were achieved by performing the IHC procedure before the SISH procedure.

Samples were deparaffinized with EZ Prep at 72°C in 6 cycles. For HER2 protein staining, the tissue sections were heat pretreated in CC1 for antigen retrieval at 95°C and then incubated with the anti-HER2 primary antibody at 37°C for 48 min after inactivation of the endogenous peroxidase using UV-inhibitor for 4 min at 37°C. The slides were incubated with a biotinylated secondary antibody followed by the application of HRP-conjugated streptavidin for 8 min. A copper enhanced DAB reaction was used to visualize the protein. For HER2 gene and CHR 17 staining, samples were heat pretreated with EZ Prep-diluted CC2 at 90°C for four cycles followed by protease digestion with ISH Protease 2 for 12 min at 37°C. The probes were denatured for 4 min at 80°C and hybridized for 6 h at 44°C using a cocktail of DNP- and DIG-labeled probes. HybClear solution was added to block the interaction between the DNP and the DAB deposit during hybridization. The gene signals were detected using rabbit anti-DNP antibody for 16 min at 37°C and incubated with a HRP-conjugated goat anti-rabbit antibody for 16 min at 37°C after four 8 min stringency washes. The signal was developed by the silver deposit with silver acetate, hydroquinone and hydrogen peroxide. For Chr 17 detection, the slides were incubated with mouse anti-DIG antibody for 16 min at 37°C and with an AP-conjugated goat anti-mouse antibody for 24 min at 37°C. The Chr 17 signal was developed as red dot staining with fast red and naphthol phosphate. Before mounting, slides were counterstained with hematoxylin II for 8 min and bluing reagent for 4 min.

### Pathology review

The samples were independently interpreted by a pathologist (AB) and a biologist trained in gastric cancer histology (DW). The observers received the samples randomly, i.e. SISH, IHC, and combined method were not evaluated in association with each other, but separately for each patient. Consensus of both reviewers was found after each evaluation. Because discordant results between both methods may be a result of intra-observer variability rather than differences in the staining quality, discordant cases were reevaluated by a third observer who was blinded to the results of the first evaluation, but evaluated the discordant cases (methods) in association with each other. Finally, consensus was established between the third observer and the primary observers.

The IHC results were interpreted using the scoring scheme proposed for gastric cancer by Hofmann et al. [17] and Rüschoff et al. [18]: 0, no reactivity or membranous reactivity in < 10% of tumor cells; 1+, faint/barely perceptible membranous reactivity in > 10% of tumor cells; 2+, weak to moderate complete, lateral or basolateral membranous reactivity in > 10% of tumor cells; and 3+, strong complete, lateral or basolateral membranous reactivity in ≥ 10% of tumor cells.

In SISH results, the total numbers of HER2 and chromosome 17 signals were counted in at least 20 tumor cell nuclei in two different areas. The HER2/Chr 17 ratios were interpreted in accordance with the ToGA FISH scoring scheme for HER2 testing in gastric and gastroesophageal junction (GEJ) cancer as follows: < 2.0, HER2 gene not amplified; ≥ 2.0, HER2 gene amplified [17]. An average count of six or more was regarded as amplified.

### Statistical analysis

The analysis was explorative with no predefined hypothesis or sample size calculations. The number of 100 patients was considered sufficient to perform the proposed statistical tests. Correlations between IHC/SISH and gene/protein were estimated using Cohen´s kappa coefficient (к). The kappa value interpretation by Altmann [19] was used: к > 0.81, very good agreement; 0.61-0.80, good agreement; 0.41-0.60 moderate agreement; 0.40-0.21, fair agreement; < 0.20, slight agreement. Statistical analyses were performed using WinStat software (R.Fitch Software, Version 2009.1).

## Results

### Patient characteristics

Ninety six of the 100 patient cases were assessable. One case was excluded because of the absence of tumor cells and three cases for poor signals in the single SISH staining.

The majority of patients were male (67.7%) and ≥ 65 years old (70.8%) (Table 1). The cohort consists of patients with adenocarcinoma of middle to distal stomach (50.0%) or GEJ (45.8%) with more cases of Lauren`s intestinal (67.7%) than diffuse tumors (28.1%). The grading of the majority of samples was G3 (50.0%) followed by G2 (39.6%) and tumors classified as G2-3 (10.4%). 85.7% of resected patients (n  =  28) showed lymph node metastasis (N stage) and 64.3% showed no distant metastases (M stage). In the half of the resected patients, T3 tumor stage was observed, followed by T2 in 28.6%, T1 in 14.3% and T4 stage in 7.1% of patients.

**Table 1:** Patient´s characteristics and HER2 positivity by subgroups

	**Total *n*** **=96 (%)**	Positivity* (IHC 3+ or SISH +) single *n * **(%)**	Positivity* (IHC 3+ or SISH +) combi *n * **(%)**
**Sex**			
Female	27 (28.1)	2 (7.4)	1 (3.7)
Male	65 (67.7)	11 (16.9)	11 (16.9)
Unknown	4 (4.2)	3 (75.0)	3 (75.0)
**Age**	18-94 yrs (Median 69)		
≥ 65	68 (70.8)	12 (17.6)	11 (16.2)
< 65	28 (29.2)	4 (14.3)	4 (14.3)
**Primary tumor location**			
Mid to distal stomach	48 (50.0)	5 (10.4)	4 (8.3)
GEJ	44 (45.8)	8 (18.2)	8 (18.2)
Not specified	4 (4.2)	3 (75.0)	3 (75.0)
**Lauren classification**			
Diffuse	27 (28.1)	3 (11.1)	2 (7.4)
Mixed	4 (4.2)	1 (25.0)	1 (25.0)
Intestinal	65 (67.7)	12 (18.5)	12 (18.5)
**Biopsy/resection**			
Biopsy	68 (70.8)	10 (14.7)	9 (13.2)
Resection	28 (29.2)	6 (21.4)	6 (21.4)
**T stage ( *n *** **=28)**			
T 1	4 (14.3)	0	0
T 2	8 (28.6)	2 (25.0)	2 (25.0)
T 3	14 (50.0)	4 (28.6)	4 (28.6)
T 4	2 (7.1)	0	0
**N stage ( *n *** **=28)**			
N +	24 (85.7)	5 (20.8)	5 (28.8)
N -	4 (14.3)	1 (25.0)	1 (25.0)
**M stage ( *n *** **=28)**			
M +	5 (17.9)	1 (20.0)	1 (20.0)
M -	18 (64.3)	3 (16.7)	3 (16.7)
Mx	5 (17.9)	2 (40.0)	2 (40.0)
**Grading**			
G2	38 (39.6)	6 (15.8)	6 (15.8)
G2-3	10 (10.4)	2 (20.0)	2 (20.0)
G3	48 (50.0)	8 (16.7)	7 (14.6)

*Abbreviations*: *IHC* immunohistochemistry, *SISH* silver in situ hybridization *T* primary tumor, *N* lymph nodes, *M* metastases *defined as IHC 3+ or SISH ≥2.0.

*P*=1 for all comparisons between IHC/SISH and gene/protein methods (Fisher´s Exact Test).

In Table 1 the HER2 positivity by subgroups is also presented. No significant difference was noted in HER2 positivity when compared to subgroups and different methods.

### Staining results for HER2 protein expression and gene amplification (single staining methods)

**Figure 1:**
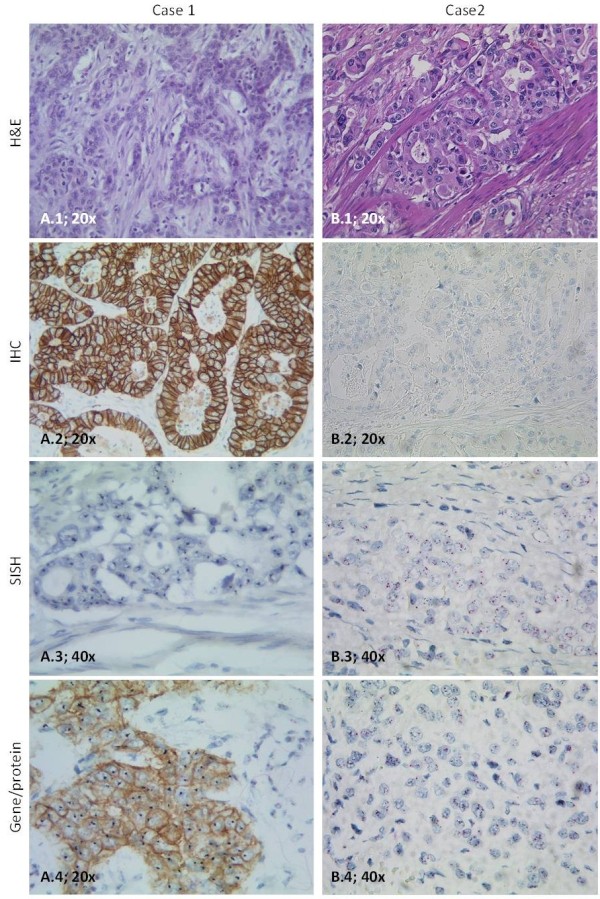
**Representative results of the different staining methods.** Intestinal tumor (hematoxylin and eosin (H&E), A.1, 20x) with IHC 3+ (immunohistochemistry (IHC), A.2, 20x; gene/protein, A.4, 20x) and amplification (silver in situ hybridization (SISH), A.3, 40x; gene/protein, A.4, 20x). IHC score 0 (IHC, B.2, 20x; gene/protein, B.4, 40x), intestinal type (H&E, B.1, 20x) and amplification (SISH, B.3, 40x; gene/protein, B.4, 40x).

Protein expression scores by IHC were 0 and 1 + in 70.8% and 10.4% of patients (Table 2). 2.9% of score 0 and 10.0% of score 1 + cases were amplified. In 9.4% of cases, a moderate expression (score 2 +) with 44.4% amplified cases was detected. 9.4% of the stained samples showed a strong expression (score 3 +) with 77.8% amplified cases. Figure 1 demonstrates representative results from single staining.

**Table 2:** Staining results for HER2 protein expression and gene amplification

	**Single method**		**Combined method**	
**IHC Score**	**IHC (*n*** **=96)**	**SISH ≥ 2.0**	**IHC (*n*** **=96)**	**SISH ≥ 2.0**
	***n *** **(%)**	***n *** **(%)**	***n *** **(%)**	***n *** **(%)**
**0**	68 (70.8)	2 (2.9)	68 (70.8)	2 (2.9)
**1+**	10 (10.4)	1 (10.0)	11 (11.5)	1 (9.1)
**2+**	9 (9.4)	4 (44.4)	7 (7.3)	2 (28.6)
**3+**	9 (9.4)	7 (77.8)	10 (10.4)	9 (90.9)

*Abbreviations*: *IHC* immunohistochemistry, *SISH* silver in situ hybridization.

### Gene/protein (combined staining method)

Protein expression scores by gene/protein method were 0, 1+, 2+, and 3+ in 70.8%, 11.5%, 7.3% and 10.4% (Table 2). There were 2.9% / 9.1% amplified cases in samples with score 0 / 1+. 28.6% amplified cases showed a moderate staining (score 2+). Samples with high expression (3+) showed in 90.9% gene amplification. Examples of gene/protein staining are demonstrated in Figure 1.

### Evaluation of Concordant and discordant cases

The assessment of IHC staining in the conventional single and the gene/protein method did not always reveal congruent results; agreements of both methods were also listed in Table 3 There were very good concordances in results of amplified cases (agreement 100.0%; Kappa-coefficient κ  =  1) as well as score 0 (100%; κ  =  1), score 1+ (98.96%; κ  =  0.947) and score 3+ tumor samples (96.88%; κ  =  0.825) in both methods. Cases with score 2+ showed less concordance, although still good (95.83%; κ  =  0.728). Concordance between the overall HER2 positivity (defined as IHC 3+ or SISH+) in standard or novel method was very good (98.69%; κ  =  0.963; 17.7% vs. 16.7%).

**Table 3:** Concordances between single methods and combined gene/protein methods

**Single vs. combination**	**Concordance**	**Kappa-Coefficient к**	**Agreement Altmann [**[15]**]**
**SISH amplified**	100 %	1	Very good
**IHC score 0**	100 %	1	Very good
**IHC score 1+**	98.96%	0.947	Very good
**IHC score 2+**	95.83%	0.728	Good
**IHC score 3+**	96.88%	0.825	Very good

*Abbreviations*: *IHC* immunohistochemistry, *SISH* silver in situ hybridization.

There were twelve discrepant cases before re-analyzing by a third observer (data not shown). After re-evaluation by a third observer, discrepant results remained in four tumor samples. The reason for the eight discrepant cases in the interim analysis was found to be intra-observer variability. Results of the final discordant results are shown in Table 4.

**Table 4:** Detailed evaluation of discordant results between methods

		**Single methods**		**Combined method**	
**Case**	**Histology (Lauren)**	**IHC (Score)**	**SISH (≥ 2.0/< 2.0)**	**IHC (Score)**	**SISH (≥ 2.0/< 2.0)**
**6693**	i	2+	≥2.0	3+	≥2.0
**9924**	i	2+	<2.0	1+	<2.0
**20209**	i	2+	≥2.0	3+	≥2.0
**1057**	d	3+	<2.0	2+	<2.0

*Abbreviations*: *IHC* immunohistochemistry, *SISH* silver in situ hybridization.

## Discussion

The aim of this study was to evaluate the feasibility of new gene/protein platform in comparison to the single staining methods in gastric adenocarcinomas and carcinomas of the esophagogastric junction.

Of the evaluable samples 28.1% showed a diffuse, 4.2% a mixed and 67.7% an intestinal histological type. The majority of patients were ≥65 years old (70.8%) and male (67.7%). The rate of adenocarcinoma of GEJ or middle to distal stomach was similar (45.8% vs. 50.0%). In the standard method, 17.7% of patients were HER2 positive. The majority of HER2-positive tumors were of intestinal histology and were localized in the GE junction, this correlates well with data of literature [20,21]. Overall, based on patient and tumor characteristics as well as HER2 positivity, the evaluated group is a representative collective.

Although considerable experience for HER2 analysis by IHC and ISH for breast cancer is available, it is not certain whether they can be directly transferred to carcinomas of the gastrointestinal tract. The expression of HER2 protein, unlike in carcinoma of the breast, is heterogeneous and often focally pronounced. Fox et al. [12]. determined the HER2 status in 100 tissue samples diagnosed with advanced stomach cancer or cancer of the esophagogastric junction in different laboratories to test their matches. For this, IHC and CISH (chromogenic in situ hybridization) or SISH were applied. There was a good agreement between the different laboratories regarding CISH or SISH, but moderate concordance for all IHC scores was observed. Based on the results of the investigation, the author's opinion was that a determination of HER2 status based on IHC, followed by ISH (European standard) is not an optimal evaluation strategy and recommended performing both staining methods [12]. Another study [22] tested the HER2 amplification and expression in 148 patients with advanced gastric carcinoma by IHC and FISH/SISH. Positive HER2 cases were detected in only 10% of the IHC compared with 18% to 22% in the in situ hybridization. Not only because it was easier to reproduce, the author proposed the in situ hybridization technique as the better approach for determining the HER2 positivity [22]. However, based on the TOGA trial [8], patients with ISH positive but IHC negative or weekly (1+) positive tumors did not benefit from trastuzumab. Therefore, the use of FISH or SISH as the primary and only method is not recommended in Europe and IHC is still considered as the primary evaluation method in many countries.

To the best of our knowledge, however, there are no studies that directly compare results of both immunohistochemistry (IHC) and silver in situ hybridization (SISH) when each is used separately with the results of using them in combination on a single section in gastric carcinomas. To visualize RNA and DNA targets in situ, there are different ways of labeling probes using fluorescence, chromogenic or silver detection. All previous studies with settings comparable to our analysis used chromogenic or fluorescence in situ hybridization (CISH or FISH). Downs-Kelly et al. [14] combined standard IHC with a deposition of metallic silver by EnzMetTM on a tissue microarray (TMA) of breast carcinomas and compared the results of IHC and fluorescence in situ hybridization (FISH) when performed alone. In another study, the authors has indeed used the combination of IHC and silver in situ hybridization (SISH) in TMA of breast and gastric cancer samples, but compared the results of those obtained by IHC and fluorescence in situ hybridization (FISH) as single staining [16]. Reisenbichler et al. [15] reported the results of the comparison of IHC and chromogenic in situ hybridization (CISH) as single stainings with the dual assay IHC/CISH. The simultaneous evaluation of protein and gene combinations on a single slide offered a highly reproducible and very robust method with good concordances.

To test the validity of the new gene/protein staining compared with the standardized IHC and SISH, the concordances were calculated based on the kappa coefficient. According to the ASCO/CAP guidelines for breast cancer, a new test should show more than 95% concordance with a validated reference assay [23]. In our study, the new method showed concordances above 95% in terms of all test scores. There were very good concordances in results of gene amplification as well as score 0 (each with 100% agreement), score 1+ (98.96% agreement) and score 3+ tumor samples (96.88% agreement). Cases with score 2+ showed less concordant results, although still good (95.83% agreement). Difficulties in cases with moderate expression in the assessment and classification are known in the literature. Tafe et al. [24]. showed that there were difficulties in assigning a final score in the evaluation of the IHC due to lack in the reliable separation of cases with a HER2 score of 1 + or 2 +. These categories might be limited by subjectivity and poor reproducibility. The phenomenon that discordant results are observed in patients with low HER2 expression, who sometimes still have HER2 gene amplification, has been reported in esophageal cancer [24-26]. The most probable reason for this discordance is intra-tumor heterogeneity of HER2 status in gastric or GEJ cancer [17,27,28]. In our study, discordant IHC results were observed in four patients, but these have led to a clinically relevant difference in HER2 positivity in one patient only (patient #1057). The patient had diffuse type gastric cancer. The evaluation of diffuse tumors seems to be more difficult than intestinal type tumors [29,30]. According to Rüschoff et al. [18]. only 5% of these tumors show HER2 positivity; signet ring cell carcinomas are negative. HER2 amplification is extremely rare in diffuse gastric carcinoma which is associated with abnormal E-cadherin expression [13,31-33].

The SISH is a relatively new method for the detection of the amplification of the HER2 gene. Numerous studies confirmed a high concordance with other in situ hybridization methods like CISH and FISH [34]. Compared to the IHC, ISH shows a higher sensitivity and specificity and a higher reproducibility. This statement can be confirmed by comparing the concordance of amplified cases in our methods. Results revealed a very good agreement between both methods (100.0%; к = 1). Furthermore, the ISH is less sensitive than IHC due to a relative stability of DNA compared to differences in tissue fixation and processing.

We are currently working on an expanded study evaluating whether this new method is associated with a reduction of inter-observer variability in the determination of HER2 status in gastric cancer. This study also includes a component looking at the time needed to report final results. The results will be submitted to the journal as letter or an original report, as soon as they are available.

## Conclusions

In this study, we have clearly shown that using the combined method to perform HER2 protein expression and gene amplification on a single slide can produce results similar to those using each method individually in a controlled study implementing three independent observers. We believe that the new gene/protein staining provides a good alternative to the conventional standard method IHC and ISH and may theoretically reduce the possibility that a gastric cancer patient will receive an incorrect HER2 status assessment and thus an incorrect treatment. This may apply especially for cases that are difficult to interpret because they have a score of 1+ or 2+, as well as in heterogeneous and diffuse cases. Moreover, the direct combination of the two staining methods can save precious patient material, since only a single tissue section is required. This has become an important tissue, as numerous biomarkers are currently under clinical evaluation for the treatment of esophagogastric cancer. Further, the staining can also be a time saving. However, it is important to note that these potential advantages are still theoretical and have to be proven in a future study.

## Abbreviations

IHC: Immunohistochemistry; SISH: Silver in situ hybridization; IHC/SISH: Single methods; gene/protein: Both methods on one slide combined; EGFR: Epidermal growth factor receptor; mTOR: Mammalian target of rapamycin; FFPE: Formalin-fixed paraffin-embedded; H&E: Hematoxylin and eosin; CC1: Cell Conditioning 1; DNP: 2,4-dinitrophenol; DIG: Digoxigenin; CC2: Cell Conditioning 2; GEJ: Gastroesophageal junction; CISH: Chromogenic in situ hybridization; FISH: Fluorescence in situ hybridization; TMA: Tissue microarray.

## Competing interests

TJ has received research funding from Roche Pharma. TL and MM have received research funding from Ventana Medical System, lnc./Roche Tissue Diagnostics. TL, MM and TJ have an employment or leadership position to Ventana Medical System, lnc./Roche Tissue Diagnostics. All other authors declare no conflicts of interests. All other authors declare that they have no competing interests.

## Authors’ contributions

DW carried out the histopathological analysis and immunohistochemistry, performed the statistical analysis and wrote the manuscript. AB and SEA carried out the histopathological analysis, designed the experiment and help to draft the manuscript. KS carried out the immunohistochemistry. TJ, TL and MM carried out the immunoassay and critically analysed the data. HMA collected tumor tissue and critically analysed the data. All authors read and approved the final manuscript.
